# Antioxidant and Nitrite-Scavenging Capacities of Phenolic Compounds from Sugarcane (*Saccharum officinarum* L.) Tops

**DOI:** 10.3390/molecules190913147

**Published:** 2014-08-26

**Authors:** Jian Sun, Xue-Mei He, Mou-Ming Zhao, Li Li, Chang-Bao Li, Yi Dong

**Affiliations:** 1 College of Light Industry and Food Sciences, South China University of Technology, Guangzhou 510640, China; E-Mail: waydongyi2501779@163.com; 2 Agro-food Science and Technology Research Institute, Guangxi Academy of Agricultural Sciences, Nanning 530007, China; E-Mails: xuemeihe1981@126.com (X.-M.H.); lili@gxaas.net (L.L.); changbaoli@gxaas.net (C.-B.L.); 3 Guangxi Crop Genetic Improvement Laboratory, Nanning 530007, China

**Keywords:** sugarcane tops, phenolic compounds, identification, antioxidantcapacity, nitrite-scavengingcapacity

## Abstract

Sugarcane tops were extracted with 50% ethanol and fractionated by petroleum ether, ethyl acetate (EtOAc), and *n*-butyl alcohol successively. Eight phenolic compounds in EtOAc extracts were purified through silica gel and Sephadex LH-20 column chromatographies, and then identified by nuclear magnetic resonance and electrospray ionization mass spectra. The results showed that eight phenolic compounds from EtOAc extracts were identified as caffeic acid, cis-*p*-hydroxycinnamic acid, quercetin, apigenin, albanin A, australone A, moracin M, and 5'-geranyl-5,7,2',4'-tetrahydroxyflavone. The antioxidant and nitrite-scavenging capacities of different solvent extracts correlated positively with their total phenolic (TP) contents. Amongst various extracts, EtOAc extracts possessed the highest TP content and presented the strongest oxygen radical absorbance capacity (ORAC), 1,1'-diphenyl-2-picrylhydrazyl (DPPH) radical-scavenging capacity, 2,2'-azobis-3-ethylbenthiaazoline-6-sulfonic acid (ABTS) radical-scavenging capacity, ferric reducing antioxidant power (FRAP) and nitrite-scavenging capacity. Thus, sugarcane tops could be promoted as a source of natural antioxidant.

## 1. Introduction

**Figure 1:**
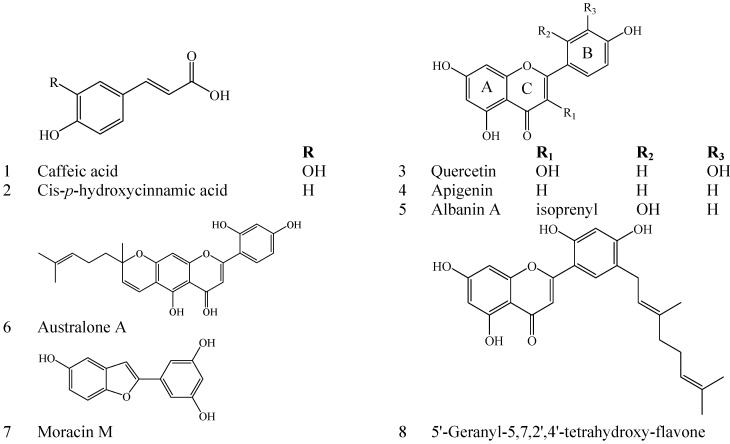
Phenolic compounds isolated from EtoAc extract of sugarcane tops.

Free radicals and other reactive oxygen species are produced by oxidative metabolism continuously *in vivo*, resulting in cell ageing, cell death, and tissue damage [1]. With increasing evidences showing the involvement of oxidative stress induced by free radicals in the development of various diseases such as skin ageing, atherosclerosis, diabetes, cancer and cirrhosis [2,3], it has been argued that excess antioxidants may impair signaling of reactive oxygen and production of lipid oxidation products [4]. Antioxidants, e.g., phenolic compounds, are beneficial for postponing ageing, preventing disorder, reducing disease risk and maintaining health by inhibition of lipid peroxidation [4,5]. The condition of oxidative stress occurs when the pro-oxidant/anti-oxidant balance change in favor of the pro-oxidant, as a result of an overproduction of free radicals, such as the peroxil, alkoxyl, and hydroxyl radicals. The increase of free radicals at cellular level leads to DNA damage, protein oxidization and lipid peroxidation, and subsequently to cell death via apoptosis or via necrosis. Convincing evidence has demonstrated that oxidative stress is involving in the physio-pathological basis of many processes, including neurodegenerative diseases, cardiovascular diseases, cancer, inflammation, and aging. Free radicals can result in genetic alterations of certain cells, thus increasing the risk of these diseases [6]. Phenolic compounds exist naturally in vegetables, fruits and grains. These compounds possess the ability to reduce oxidative damage because they can act as direct antioxidant by donating a hydrogen atom to free radicals and by chelating metal ions, such as iron or copper, as well as they can act as indirect antioxidants by upregulating antioxidant enzymes [6,7]. These antioxidant properties of phenolic compounds are directly related to their chemical structure, and particularly to the phenol group [6]. Phenolic compounds are of interest in pharmaceutical and food industries. Their pharmacological actions are ascribed to the free radical scavenging and metal ion chelating activities, and their effects on pathways of cell-signaling and on gene expression [8]. The antioxidant capacities of phenolic compounds are often assessed by the Trolox equivalent antioxidant capacity (TEAC), the ferric reducing antioxidant power (FRAP), the hypochlorite scavenging capacity, the deoxyribose method and the copper-phenanthroline-dependent DNA oxidation assays. The multidimensional efficacies of phenolic compounds differ depending on the mechanism of antioxidant action in diverse experimental systems. Synthetic phenolic compounds, e.g., butylated hydroxyanisole (BHA), have been restricted in food industry for carcinogenic possibility. Hence, natural antioxidants have been extensively investigated in recent years [9]. Sugarcane (*Saccharum officinarum* L.) is one of the important crops in subtropical and tropical areas. Some varieties are consumed as fruits. Sugarcane is a principal sugar source for the food industry. Approximately 70% of the sugar produced globally comes from sugarcane. Sugarcane tops, yielding about 15% of the total sugarcane yield [10], are good sources for natural antioxidants. They are often thrown away, left to rot, burnt, used as forage, or produced into beverages. It was reported recently that sugarcane tops possessed higher flavonoid contents than sugarcane stems [11]. Although sugarcane tops contain plentiful phenolic compounds, they have still not been developed and utilized adequately up until now. We reported herein the isolation and structural identification of eight phenolic compounds (Figure 1) in sugarcane tops. Oxygen radical absorbance capacity, DPPH radical-scavenging capacity, ABTS radical-scavenging capacity, ferric reducing antioxidant power and nitrite-scavenging capacity of different extracts from sugarcane tops were also determined. The results obtained will be useful for indicating potential bioactivities and health benefits of sugarcane tops which can be utilized as a new source of natural antioxidant in the food industry. These data are also important to illuminate that sugarcane top phenolics possess an antioxidant protection role, which can postpone ageing and senescing.

**Figure 2:**
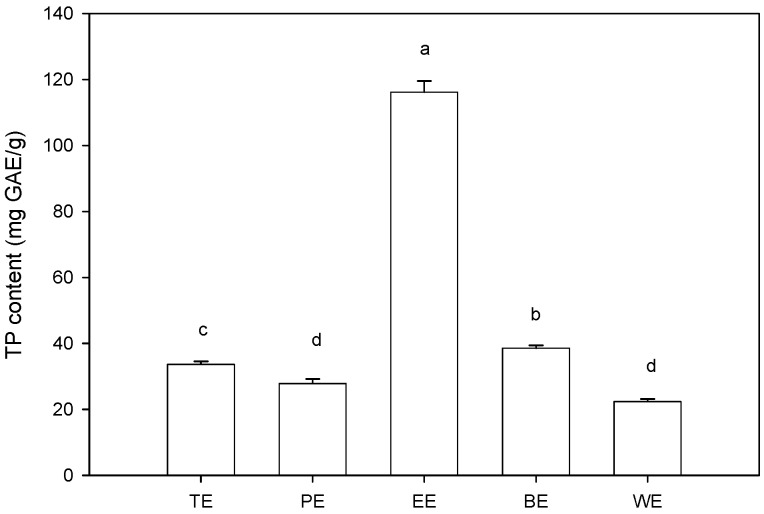
Total phenolic content in different extracts of sugarcane tops. TE, PE, EE, BE and WE were the abbreviations of total extract, petroleum ether extract, EtOAc extract, *n*-BuOH extract, and water extract, respectively.Different letters horizontally (a–d) indicated significant differences (*p* < 0.05) of total phenolic content among different extracts according to Duncan’s multiple range test.

## 2. Results and Discussion

### 2.1. Total Phenolic Content

TP contents of various extracts from sugarcane tops are shown in Figure 2. Statistical analysis indicated that TP contents were significantly (*p* < 0.05) affected by the difference of extracting solvent. TP contents ranged from 22.33 ± 0.83 to 116.17 ± 3.40 mg GAE/g extracts, and the order was as follows: EtOAc extract > *n*-BuOH extract > total extract > petroleum ether extract > water extract. The highest TP content of EtOAc extract indicated that phenolic components in sugarcane tops mainly dissolved in EtOAc, which was the optimum solvent for separating and enriching phenolic compounds from sugarcane top extracts.

### 2.2. Identification of Major Phenolic Compounds in Sugarcane Tops

Eight phenolic compounds (Figure 1) were isolated from EtOAc extract with the highest TP content. Their chemical structures were identified as follows:

Compound **1**: pale yellow powder, mp 208–209 °C, ESI-MS *m/z*: 179 [M−H]^–^. ^1^H-NMR (DMSO-*d*_6_, 400 MHz) δ: 9.6 (1H, s, H-9), 7.44 (1H, d, *J =* 16.0 Hz, H-7), 7.08 (1H, d, *J =* 3.0 Hz, H-2), 6.99 (1H, dd, *J =* 3.0, 9.2 Hz, H-6), 6.65 (1H, d, *J =* 9.0 Hz, H-5), 6.17 (1H, d, *J =* 16.0 Hz, H-8); ^13^C-NMR (DMSO-*d*_6_, 100 MHz) δ: 126.0 (C-1), 115.1 (C-2), 145.6 (C-3), 148.3 (C-4), 115.1 (C-5), 121.0 (C-6), 144.4 (C-7), 115.9 (C-8), 168.3 (C-9). Compound **1** was identified as caffeic acid by comparing NMR and MS data with literature [12].

Compound **2**: white needles (H_2_O), mp 131–133 °C, ESI-MS *m/z*: 164 [M+H]^+^, 147, 138, 121, 119, 93, 65. IR (KBr) ν_max_: 3400, 1675, 1606, 1511, 1445, 1240, 1211, 988 cm^–1^. ^1^H-NMR (DMSO-*d*_6_, 400 MHz) δ: 7.66 (2H, d, *J =* 7.4 Hz, H-2, 6), 6.70 (2H, d, *J =* 7.4 Hz, H-3, 5), 6.62 (1H, d, *J =* 12.0 Hz, H-7), 5.68 (1H, d, *J =* 12.0 Hz, H-8); ^13^C-NMR (DMSO-*d*_6_, 100 MHz) δ: 126.1 (C-1), 132.5 (C-2, 6), 115.0 (C-3, 5), 158.8 (C-4), 140.7 (C-7), 118.4 (C-8), 168.5 (C-9). Compound **2** was confirmed as cis-*p*-hydroxycinnamic acid by NMR and MS data with literature [13].

Compound **3**: yellow needles (MeOH), mp 308–309 °C, UV (MeOH) λ_max_ (log ε): 258, 379. IR (KBr) ν_max_: 3420, 3370, 3289, 1672, 1615, 1520, 1432, 1360 cm^–1^. ESI-MS *m/z*: 301 [M−H]^–^. ^1^H-NMR (DMSO-*d*_6_, 400 MHz) δ: 12.49 (1H, s, OH-5), 10.76 (1H, s, OH-7), 9.58 (1H, s, OH-4'), 9.35 (1H, s, OH-3'), 9.30 (1H, s, OH-3), 7.68 (1H, d, *J =* 1.5 Hz, H-2'), 7.55 (1H, dd, *J =* 9, 1.5 Hz, H-6'), 6.89(1H, d, *J =* 9 Hz, H-5'), 6.41(1H, d, *J =* 2 Hz, H-6), 6.19(1H, d, *J =* 2 Hz, H-6); ^13^C-NMR (DMSO-*d*_6_, 100 MHz) δ: 146 (C-2), 136 (C-3), 176(C-4), 103.3 (C-4a), 157 (C-5), 98.1 (C-6), 164.4 (C-7), 93.2 (C-8), 163.3 (C-8a), 123.2 (C-1'), 114.9 (C-2'), 145.6 (C-3'), 147.3 (C-4'), 114.3 (C-5'), 121.2 (C-6'). Compound **3** was identified as quercetin by the comparison of NMR and MS data with literature [14].

Compound **4**: yellow needles (MeOH), mp 340–342 °C, ESI-MS *m/z*: 269 [M−H]^–^. IR (KBr) ν_max_: 3288, 1655, 1606, 1510 cm^–1^. ^1^H-NMR (DMSO-*d*_6_, 400 MHz) δ: 7.99 (2H, dd, *J =* 8.4, 2.4 Hz, H-2', 6'), 6.94 (2H, dd, *J =* 8.4, 2.4 Hz, H-3', 5'), 6.88 (1H, s, H-3), 6.56 (1H, d, *J =* 2.4 Hz, H-8), 6.21 (1H, d, *J =* 2.4 Hz, H-6); ^13^C-NMR (DMSO-*d*_6_, 100 MHz) δ: 164.1 (C-2), 103.1 (C-3), 181.6 (C-4), 157.7 (C-4a), 162.0 (C-5), 99.8 (C-6), 164.0 (C-7), 94.3 (C-8), 104.1 (C-8a), 121.1 (C-1'), 128.2 (C-2', 6'), 116.4 (C-3', 5'), 161.5 (C-4'). Compound **4** was identified as apigenin by comparing NMR and MS data with literature [15].

Compound **5**: yellow powder, ESI-MS *m/z*: 353 [M−H]^–^. ^1^H-NMR (DMSO-*d*_6_, 400 MHz) δ: 13.13 (1H, br s, 5-OH), 7.18 (1H, d, *J =* 8.4 Hz, H-6'), 6.56 (1H, d, *J =* 2.4 Hz, H-3'), 6.51 (1H, dd, *J =* 8.4, 2.4 Hz, H-5'), 6.31(1H, d, *J =* 3.2 Hz, H-8), 6.23 (1H, d, *J =* 3.2 Hz, H-6), 5.11 (1H, t, *J =* 5.6 Hz, H-10), 3.1 (2H, d, *J =* 6.8 Hz, H-9), 1.56 (3H, s, H-13), 1.42 (3H, s, H-12); ^13^C-NMR (DMSO-*d*_6_, 100 MHz) δ: 161.5 (C-2), 121.6 (C-3), 182.9 (C-4), 105.0 (C-4a), 163.3 (C-5), 99.4 (C-6), 165.3 (C-7), 94.3 (C-8), 159.3 (C-8a), 24.6 (C-9), 122.7 (C-10), 132.0 (C-11), 25.8 (C-12), 17.6 (C-13), 113.0 (C-1'), 157.2 (C-2'), 103.9 (C-3'), 162.3 (C-4'), 108.1 (C-5'), 132.2 (C-6'). Compound **5** was identified as albanin A by comparing NMR and MS data with literature [16].

Compound **6**: yellow power, ESI-MS *m/z*: 419 [M−H]^–^. ^1^H-NMR (Acetone-*d*_6_, 400 MHz) δ: 7.85 (1H, d, *J =* 8.8 Hz, H-6'), 6.62 (IH, d, *J =* 1.7 Hz, H-3'), 6.55(1H, dd, *J =* 8.8, 1.7 Hz, H-5'), 6.71 (1H, d, *J =* 10 Hz, H-9), 5.71 (1H, d, *J =* 10 Hz, H-10), 6.46 (1H, s, H-8), 5.13 (1H, t, *J =* 7.2 Hz, H-15), 2.1–2.2 (1H, m, H-14), 1.6-1.8 (1H, m, H-13), 1.64 (3H, s, H-12), 1.57 (3H, s, H-17), 1.45 (6H, s, H-18); ^13^C-NMR (Acetone-*d*_6_, 100 MHz) δ: 162.6 (C-2), 108.5 (C-3), 183.4 (C-4), 105.6 (C-4a), 156.9 (C-5), 105.7 (C-6), 159.6 (C-7), 95.3 (C-8), 158.0 (C-8a), 116.4 (C-9), 128.0 (C-10), 81.1 (C-11), 27.0 (C-12), 42.2 (C-13), 23.7 (C-14), 124.8 (C-15), 132.2 (C-16), 18.0 (C-17), 25.7 (C-18), 110.6 (C-1'), 160.3 (C-2'), 104.3 (C-3'), 163.0 (C-4'), 109.0 (C-5'), 130.9 (C-6'). Compound **6** was identified as australone A by comparing NMR and MS data with literature [17].

Compound **7**: brown powder, ESI-MS *m/z*: 241 [M−H]^–^. ^1^H-NMR (DMSO-*d*_6_, 400 MHz) δ: 7.38 (1H, d, *J =* 8.4 Hz, H-4), 7.06 (1H, s, H-3), 6.92 (1H, d, *J =* 2.0 Hz, H-7), 6.74 (1H, dd, *J =* 8.4, 2.0 Hz, H-5), 6.67 (2H, d, *J =* 3.2 Hz, H-2',6'), 6.22 (1H, t, *J =* 3.2 Hz, H-4'); ^13^C-NMR (DMSO-*d*_6_, 100 MHz) δ: 153.9 (C-2), 101.5 (C-3), 121.0 (C-3a), 120.7 (C-4), 112.4 (C-5), 155.8 (C-6), 97.4 (C-7), 155.2 (C-7a), 131.6 (C-1'), 102.3 (C-2'), 158.8 (C-3'), 102.6 (C-4'), 158.8 (C-5'), 102.3 (C-6'). Compound **7** was identified as moracin M by comparing NMR and MS data with literature [18]. 

Compound **8**: yellow powder, UV (MeOH) λ_max_ (log ε): 216.2 (0.85), 257.0 (0.51), 289.4 (0.29), 361.6 (0.59). IR (KBr) ν_max_: 3427, 2929, 1621, 1492, 1278, 1151, 1122, 1060, 968, 822 cm^–1^. ESI-MS *m/z*: 421.3 [M−H]^–^. ^1^H-NMR (Acetone-*d*_6_, 400 MHz) δ: 13.10 (1H, br s, 5-OH), 7.70 (1H, s, H-6'), 7.10 (1H, s, H-3), 6.66 (1H, s, H-3'), 6.46 (IH, d, *J =* 2 Hz, H-8), 6.23 (1H, d, *J* = 2 Hz, H-6), 5.39 (1H, t, *J* = 7.3 Hz, H-2''), 5.13 (1H, t, *J* = 6.0 Hz, H-7''), 3.32 (2H, d, *J* = 7.6 Hz, H-1''), 2.14 (2H, m, H-6''), 2.06 (2H, m, H-5''), 1.76 (3H, s, H-4''), 1.60 (3H, s, H-10''), 1.57 (3H, s, H-9''); ^13^C-NMR (Acetone-*d*_6_, 100Hz) δ: 163.2 (C-2), 104.2 (C-3), 183.4 (C-4), 105.0 (C-4a), 158.9 (C-5), 99.5 (C-6), 165.1 (C-7), 94.6 (C-8), 160.1 (C-8a), 110.3 (C-1'), 157.5 (C-2'), 108.4 (C-3'), 163.2 (C-4'), 121.4 (C-5'), 130.2 (C-6'), 28.1 (C-1''), 123.6 (C-2''), 136.5 (C-3''), 16.2 (C-4''), 40.5 (C-5''), 27.5 (C-6''), 125.0 (C-7''), 131.8 (C-8''), 25.8 (C-9''), 17.8 (C-10''). Compound **8** was identified as 5'-geranyl-5,7,2',4'-tetrahydroxy-flavone by comparing NMR and MS data with literature [16].

### 2.3. Antioxidant Capacity

#### 2.3.1. ORAC Assay

**Figure 3:**
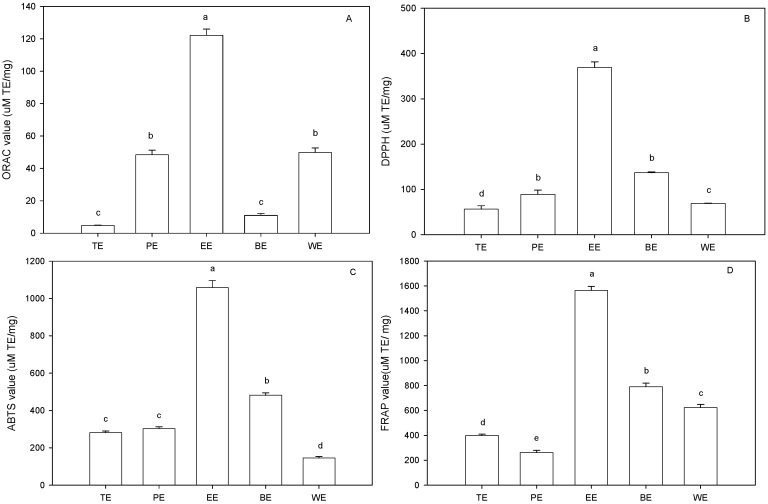
Antioxidant abilities of different extracts of sugarcane tops. ORAC assay (**A**), DPPH radical-scavenging capacities assay (**B**), ABTS radical-scavenging capacities assay (**C**) and FRAP assay (**D**). TE, PE, EE, BE, and WE were the abbreviations of total extract, petroleum ether extract, EtOAc extract, n-BuOH extract and water extract, respectively.Different letters horizontally (a–e) indicated significant differences (*p* < 0.05) of antioxidant abilities among different extracts according to Duncan’s multiple range test.

The antioxidant activity of phenolic compounds and their metabolites *in vitro* depends upon the arrangement of functional groups about nuclear structure. Sufficient evidence support that the role of specific structural components are requisites for free radical scavenging, metal chelation and oxidant activity [19]. Both configuration and total number of hydroxyl groups substantially influence several mechanisms of antioxidant activity. ORAC assay is a recent but widely accepted analysis method for “total” antioxidant capability because it is more sensitive and effective than other methods. ORAC values are also used as standard measures for comparing antioxidant activity of food materials [20,21]. Total antioxidant potential of five sugarcane top extracts was measured by ORAC assay (Figure 3A). Statistical analysis indicated that the category of extracting solvent significantly affected ORAC values which varied from 4.76 ± 1.09 μM TE/mg to 122.18 ± 3.8 μM TE/mg. EtOAc extract possessed the highest (*p* < 0.05) ORAC value, followed by water extract, petroleum ether extract, *n*-BuOH extract, and total extract. EtOAc extract exhibiting the highest TP content and ORAC value (Figure 2 and Figure 3A) suggested that ORAC value was positively related to TP content. This ratiocination was confirmed through Table 1, in which the correlation coefficient of TP content and ORAC was 0.824.

**Table 1:** Correlation analysis of total phenolic content and antioxicant abilities.

TP Content	ORAC	FRAP	DPPH Scavenging Capacities	ABTS Scavenging Capacities	Nitrite-Scavenging Capacities
Correlation coefficient	0.824	0.926	0.982	0.979	0.971
Significance (bilateral)	0.086	0.024	0.003	0.004	0.006

#### 2.3.2. DPPH and ABTS Radical Scavenging Capacities

Oxidative damage caused by free radicals may be related to aging and diseases such as atherosclerosis, diabetes, cancer, and cirrhosis. Phenolic compounds can efficiently catch hydrogen atom and provide hydrogen atom to free radicals through phenolic oxhydryl, and then phenolic compounds transform into stable phenoxy radical to inhibit oxidant development [22,23]. Total free radical-scavenging capacities of five extracts from sugarcane tops were measured using commercially available stable free radicals DPPH (Figure 3B) and ABTS (Figure 3C scavenging ability, followed by *n*-BuOH extract, petroleum ether extract, water extract, and total extract. DPPH radical scavenging values varied from 56.2 ± 7.7 μM TE/mg to 369.2 ± 12.4 μM TE/mg. The order of ABTS radical-scavenging capacity was EtOAc extracts > *n*-BuOH extracts > petroleum ether extracts > total extracts > water extracts. Both DPPH and ABTS radical-scavenging capacities were significantly (*p* < 0.05) correlated with TP content (Table 1), indicating that the main chemical substances for scavenging free radical in sugarcane tops were phenolic components. The similar result was found in sugarcane juice by Kadam *et al.* [24] who reported that EtOAc extract from sugarcane juice exhibited the high TP content and the strong free radical scavenging capacity. Phenolic components from sugarcane tops, containing more hydroxyl groups, exhibited very high ability to scavenge DPPH and ABTS radicals. The chemistry of phenolic components from sugarcane tops is predictive of their free radical scavenging activity because the reduction potentials of phenolic radicals are lower than those of DPPH and ABTS radicals, implying that phenolic compounds may not only deactivate these oxyl species, but also inhibit deleterious consequences of the reactions [25].

#### 2.3.3. FRAP Assay

In FRAP assay, antioxidant power refers as “reducing ability” which is measured by potassium ferricyanide reduction method. EtOAc extract had the highest FRAP value, followed by *n*-BuOH extract, water extract, total extract, and petroleum ether extract (Figure 3D). FRAP value varied from 261.41 ± 17.6 μM TE/mg to 1564.77 ± 32.3 μM TE/mg. FRAP value was significantly (*p* < 0.05) correlated with TP content (Table 1). It was reported that reducing ability was generally associated with the presence of reductones. The antioxidant action of reductones was based on the breaking of the free-radical chain by donating a hydrogen atom. The phenolic components of sugarcane tops extract may act as reductones by donating electrons, reacting with free radicals to convert them to more stable products and terminating the free radical chain reaction [26].

### 2.4. Nitrite-Scavenging Capacity

**Figure 4:**
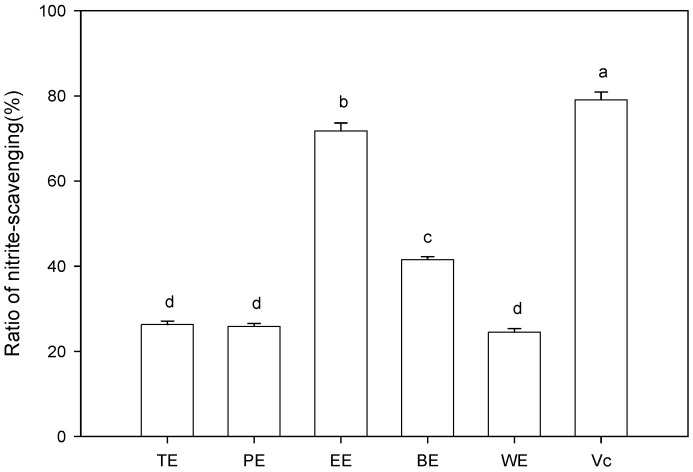
Nitrite-scavenging capacity of different extracts of sugarcane tops. TE, PE, EE, BE, and WE were the abbreviations of total extract, petroleum ether extract, EtOAc extract, *n*-BuOH extract, and water extract, respectively. Different letters horizontally (a–d) indicated significant differences (*p* < 0.05) of nitrite-scavenging capacity among different extracts according to Duncan’s multiple range test.

Nitrite-scavenging capacities of different extract from sugarcane tops are shown in Figure 4. Statistical analysis showed that the category of extracting solvent significantly influenced nitrite-scavenging capacity. EtOAc extract presented the highest (*p* < 0.05) nitrite-scavenging ratio among five extract, followed by *n*-BuOH extract, total extract, petroleum ether extract, and water extract. The nitrite-scavenging ratio of EtOAc extract approached to that of vitamin C, indicating that EtOAc extract of sugarcane tops was probably a good nitrite-scavenging additive for the food industry. From Table 1, nitrite-scavenging capacity was significantly related to TP content (*p* < 0.05), suggesting that phenolic components played an important role in scavenging nitrite. This finding was similar to the previous report by Liu *et al.* [27], who found that the flavonoid-enriched extract of *Maydis stigma* had significant scavenging ability on nitrite. Phenolic compounds could inhibit the formation of *N*-nitroso-dimethylamine due to their hydroxyl groups. At low pH, the nitrite was converted to nitrous acid and subsequently to N_2_O_3_, which could be rapidly reduced to NO by phenolic compounds that existed in sugarcane tops. Consequently, the formation of N-nitrosamine was inhibited, and the nitrite was also scavenged.

## 3. Experimental Section

### 3.1. Plant Materials

Sugarcane variety of “ROC22” (one of mainly cultivated variety in China) was chosen as plant material. Sugarcane materials were obtained from a cane experimental base in Guangxi Sugarcane Research Institute, Guangxi Academy of Agricultural Sciences on 20 November, 2011. The healthy sugarcane tops were cut, sun-dried and crushed into powder. The dried powdered plant material was stored at room temperature (25 °C) in a desiccator until further analysis.

### 3.2. Chemicals and Reagents

Ascorbic acid, ABTS diammonium salt, DPPH, fluorescein (FL), Folin–Ciocalteu reagent, trolox (6-hydroxy-2,5,7,8-tetramethylchroman-2-carboxylic acid), and 2,2'-azobis (2-amodinopropane) dihydrochloride (AAPH) were purchased from Sigma Chemical Co. (St. Louis, MO, USA). Other reagents were of analytical grade. 

### 3.3. Preparation of Sugarcane Tops Extracts

The dry powder of sugarcane tops (4.1 kg) was extracted with 50% ethanol (EtOH) with the solid/liquid rate of 1/10 (m:v) for 90 min at 70 °C. EtOH extract was evaporated to dryness by a RE52AA rotary evaporator (Yarong Equipment Co., Shanghai, China) under reduced pressure at 50 °C. The residue was dissolved in water and fractionated by petroleum ether. Petroleum ether-soluble fraction was collected and the residue was extracted by ethyl acetate (EtOAc). EtOAc-soluble fraction was collected and the residue was further extracted n-butyl alcohol (*n*-BuOH). Petroleum ether-soluble fraction, EtOAc-soluble fraction, *n*-BuOH-soluble fraction, and water-soluble fraction were obtained. All the fractions were concentrated separately under reduced pressure and freeze-dried to get petroleum ether extract, EtOAc extract, *n*-BuOH extract, and water extract. The crude 50% EtOH extract was expressed as total extract. Five different extracts (1 mg) were precisely weighed, dissolved in 1 mL of 50% EtOH, and then diluted for further analysis.

### 3.4. Determination of Total Phenolic Content

Total phenolic (TP) content in sugarcane tops extracts was measured by the method of Dorman *et al.* [28]. TP content was calculated according to the standard curve for gallic acid solutions and expressed as milligrams of gallic acid equivalents per gram different sugarcane tops extracts (mg GAE/g).

### 3.5. Isolation and Identification of Major Phenolic Compounds

EtOAc extract (38 g, this extract possessed the highest TP content) from sugarcane tops was purified using silica gel column chromatography (900 g, 100–200 mesh), eluting with gradient mixtures of chloroform-methanol (100:0–100:100, v/v) to obtain 10 fractions (A–J). Fraction C (8 g) was separated by silica gel column (100 g, 200–300 mesh) using petroleum ether-acetone (100:0–70:30, v/v) as eluents to afford 8 fractions (C1–C8). Fraction C2 and C5 was further subjected to Sephadex LH-20 column chromatography eluting with methanol to afford compound **1** (4 mg) and compound **2** (6 mg). Fraction D (3.4 g) was purified using Sephadex LH-20 column eluting with chloroform-methanol (1:1, v/v) to give compound **3** (5 mg), compound **4** (7 mg) and compound **5** (5 mg). Fraction E (7.2 g) was subjected to silica gel column chromatography (100 g, 200–300 mesh) eluting with chloroform-methanol (9:1, v/v) to give 7 fractions (E1–E7). Fraction E2 (1.2 g) was further purified using Sephadex LH-20 column eluting with methanol to give compound **6** (8 mg), compound **7** (5 mg), and compound **8** (4 mg).

The chemical structures of isolated phenolic compounds were identified by nuclear magnetic resonance (NMR) spectroscopy, electrospray ionization mass spectra (ESI-MS), and infrared (IR) spectroscopy. NMR spectra were measured on a Bruker AV-400 spectrometer (Bruker Biospin, Rheinstetten, Germany) (^1^H-NMR, 400 MHz; ^13^C-NMR, 100 MHz). Chemical shift values (δ) were recorded in parts per million (ppm) relative to tetramethylsilane (TMS) as an internal standard. ESI-MS spectra were recorded on a Finnigan LCQ Advantage Max ion trap mass spectrometer (Finnigan MAT, San Jose, CA, USA). IR spectra were determined on a Jasco FT/IR-480 plus Fourier transform (Jasco, Tokyo, Japan). Melting points (mp) were recorded on a XT-4 micro melting point apparatus (Beijing Tech Instrument Co. Ltd, Beijing, China) without correction.

### 3.6. Antioxidant and Nitrite-Scavenging Capacity Assays

#### 3.6.1. Oxygen Radical Absorbance Capacity

ORAC was determined by the modified methods of Cao and Prior [29], Ana *et al.* [30], and Lin *et al.* [31]. The assay was carried out on a Varioskan Flash spectral scan multimode plate reader (Thermo Fisher Scientific, Thermo Electron Co., Waltham, MA, USA), using 96-well polystyrene white microplates with an excitation wavelength of 485 nm and an emission wavelength of 530 nm. The fluorescein sodium salt (FL) stock solution (39.9 μM) was kept at 4 °C in the dark, and was diluted to 0.159 μM fresh FL working solution. A total of 0.2593 g of AAPH was accurately weighed and made into a 38.25 mM solution, which was kept in an ice bath. All above chemicals were dissolved in 75 mM sodium phosphate buffer (pH 7.4). A total of 25 μL of sample (different solvent extracts) and 25 μL of 75 mM sodium phosphate buffer (blank) were added in a well of 96-well microtitre, and then 75 μL of FL working solution was added. The mixture was preincubated for 10 min at 37 °C. Finally, 100 μL of AAPH solution was added rapidly. The microplate was placed in the reader and automatically shaken prior to each reading. Fluorescence was measured every minute for 70 min. Trolox solution (0.5–2.5 μM) was used as positive control and measured in every assay. ORAC value was quantified using the regression equation between Trolox concentration and net area under curve (AUC).

The ORAC values, expressed as μM trolox equivalents (μM TE/mg) were calculated by applying the following formula:

AUC = 0.5(*f*_0_ + *f*_n_) + (*f*_1_ + *f*_2_ + …… + *f*_i_ + …… + *f*_n–1_)

where *f*_0_ was initial FL reading at time 0 and *f*_i_ was FL reading at time i. Net AUC was calculated as AUC_sample_ − AUC_blank_.

#### 3.6.2. DPPH Radical-Scavenging Capacity

DPPH radical-scavenging capacity was determined according to the modified method of Luo *et al.* [32] and expressed as Trolox equivalent. Two milliliters of sample were added into 2 mL of 0.2 mM DPPH solution (in 50% ethanol). The absorbance at 517 nm of the mixture was measured after 30 min of incubation at 25 °C. The absorbance of blank and control were obtained by replacing DPPH solution or sample with ethanol, respectively. DPPH radical scavenging ratio (%) was calculated as [1 − (Asample − Ablank)/Acontrol] × 100. Where, A_sample_ was absorbance of sample, A_blank_ was absorbance of blank and A_control_ was absorbance of control. DPPH radical scavenging value was determined using a standard curve of Trolox (0.05–0.3 mM) and the results were expressed as micromolar Trolox equivalent per milligram extracts (μM TE/mg).

#### 3.6.3. ABTS Radical-Scavenging Capacity

ABTS radical-scavenging capacity was determined according to the modified method of Roberta *et al.* [33]. This method was based on the capacity of antioxidant to inhibit ABTS radical cation (ABTS^+^) compared with Trolox. ABTS^+^ stock solution was produced by dissolving ABTS in 2.45 mM potassium persulfate solution and keeping the mixture in the dark for 12–16 h at room temperature. The stock solution was diluted using 10 mM sodium phosphate buffer (pH 7.4) to obtain the working solution with an absorbance of 0.7 at 734 nm. A total of 40 μL of sample was mixed with 4 mL of ABTS·^+^ working solution for 30 s before measurement at 734 nm. ABTS^+^ scavenging ratio (%) was calculated as (1 − A_sample_/0.7) × 100. ABTS^+^ scavenging value was determined using a standard curve of Trolox (0–1 mM) and the results were expressed as micromolar Trolox equivalent per milligram extracts (μM TE/mg).

#### 3.6.4. Ferric-Reducing Antioxidant Power

FRAP was conducted according to the modified method of Jayaprakasha *et al.* [34]. One milliliter of sample, 2.5 mL of 0.2 M sodium phosphate buffer (pH 6.6) and 2.5 mL of 1% potassium ferricyanide were mixed in a 10 mL test tube. After incubation for 20 min at 50 °C, 2.5 mL of 10% trichloroacetic acid was added into the mixture. Two milliliters of upper layer were taken out and mixed with 2 mL of distilled water and 0.1% ferric chloride. The absorbance was measured at 700 nm after 10 min. A high absorbance of reaction mixture indicated a strong reducing power. The reducing power value of sample was expressed as micromolar Trolox equivalent per milligram extracts (μM TE/mg).

#### 3.6.5. Nitrite-Scavenging Capacity Assay

Nitrite-scavenging capacity was evaluated by the method of hydrochloric acid naphthalene ethylenediamine coloration. One milliliter of sample or 1 mL of 50% ethanol (blank) was mixed with 1 mL of 5 mg/L nitrite solution and 1 mL of citric acid buffer (pH 3). After reacting for 30 min at 37 °C, 1 mL of 4 g/L amino benzene sulfonic acid sodium (in 20% hydrochloric) was added in the mixture, and then 0.5 mL of 2 g/L hydrochloric acid naphthalene ethylenediamine (in water) was also added after 3 min. The mixture was reacted for 15 min and measured at 538 nm. Ascorbic acid was used as positive control. Nitrite-scavenging ratio (%) = (1 − A_sample_/A_blank_) × 100.

### 3.7. Statistical Analysis

All experiments were performed in triplicate (n = 3) and the results were expressed as mean ± standard deviation. Statistical analysis was carried out by SPSS 18 statistical software (SPSS Inc., Chicago, IL, USA). A difference was considered statistically significant when *p* < 0.05.

## 4. Conclusions

Eight phenolic compounds were isolated from EtOAc extract of sugarcane tops. The effects of different extracts (*i.e.*, total extract, petroleum ether extract, EtOAc extract, *n*-BuOH extract and water extract) on biological activities (antioxidant and nitrite scavenging capacities) were investigated *in vitro*. Amongst them, EtAOc extract from sugarcane tops possessed the strongest antioxidant and nitrite scavenging capacities. Further investigation will be performed to study the correlation between antioxidant activity and chemical structure of sugarcane top phenolics. Antioxidant and nitrite scavenging capacities of individual phenolic compounds from sugarcane tops will be determined in following research. The functional products will be also explored from sugarcane tops.
